# Supplementary material for: MetaRibo-Seq measures translation in microbiomes
Source: Nat Commun. 2020 Jun 29;11:3268. doi: 10.1038/s41467-020-17081-z (PMC7324362; doi:10.1038/s41467-020-17081-z)
Supplement: Supplementary file 10 — Supplementary Data 7 [file 41467_2020_17081_MOESM10_ESM.zip › File2/Confidence_High_Taxonomy/90574_out.krona.html]

Javascript must be enabled to view this page.

members
magnitude
magnitudeUnassigned
count
unassigned
taxon
rank

90574\_out

6

6
2
superkingdom

6
phylum
201174

6
class
1760

1
order
85006

family
1268
1

1
32207
genus

1739485
species
1

SRS013949\_contig\_number\_637

5
2037
order

5
family
2049

1654
genus
5

2

SRS012916\_contig\_number\_1356SRS143423\_contig\_number\_13568
712118
species


SRS019127\_contig\_number\_760SRS1041114\_contig\_number\_10742SRS893291\_contig\_number\_5098
3
55565
species
